# Usefulness of a Fourth Generation ELISA Assay for the Reliable Identification of HCV Infection in HIV-Positive Adults from Gabon (Central Africa)

**DOI:** 10.1371/journal.pone.0116975

**Published:** 2015-01-24

**Authors:** François Rouet, Luc Deleplancque, Berthold Bivigou Mboumba, Jeanne Sica, Augustin Mouinga-Ondémé, Florian Liégeois, Alain Goudeau, Frédéric Dubois, Catherine Gaudy-Graffin

**Affiliations:** 1 Laboratoire de Rétrovirologie, Centre International de Recherches Médicales de Franceville (CIRMF), Franceville, Gabon; 2 Centre de Traitement Ambulatoire (CTA), Franceville, Gabon; 3 UMI 233 « Trans VIH MI » (Transitions épidémiologiques, recherches translationnelles appliquées au VIH et aux Maladies Infectieuses), Institut de Recherche pour le Développement (IRD) et Université de Montpellier 1 (UM1), Montpellier, France; 4 Service de Bactériologie-Virologie, Hôpital Bretonneau, CHRU Tours, France; 5 INSERM U966, Université François Rabelais, PRES Centre Val de Loire Université, Tours, France; 6 Institut inter régional pour la Santé (IRSA), La Riche, France; University of North Carolina School of Medicine, UNITED STATES

## Abstract

**Background/Objectives:**

Guidelines for optimized HCV screening are urgently required in Africa, especially for patients infected with HIV, who sometimes show false positive or false negative reactivity in anti-HCV antibody assays. Here, we assessed the usefulness of a fourth-generation HCV Ag-Ab ELISA for the identification of active HCV infection in HIV-positive patients.

**Methods:**

This cross-sectional study was conducted between 03/2010 and 01/2013 and included 762 Gabonese HIV-positive adult patients. The results of ELISA (Monolisa HCV Ag-Ab ULTRA, Bio-Rad) were compared with those obtained by RT-PCR (gold standard). The optimal ELISA signal-to-cutoff (S/CO) ratio to identify patients with active hepatitis C (positive HCV RNA) was determined. Specimens were further tested by the INNO-LIA HCV Score assay (Innogenetics) and the Architect HCV Ag kit (Abbott) to define the best diagnostic strategy.

**Results:**

Sixty-seven patients tested positive for HCV (S/CO ratio ≥ 1) by ELISA. Of these, 47 (70.1%) tested positive for HCV RNA. The optimal S/CO associated with active HCV infection was 1.7. At this threshold, the sensitivity of ELISA was 97.9% (95% confidence interval (CI) 90.0–99.9%), its specificity was 91.3% (95% CI 85.0–95.5%), and HCV seroprevalence rate was 7.3% (56/762) (95% CI 5.6–9.4%). Among 57 HCV-seropositive patients with available INNO-LIA results, false reactivity was identified in 14 (24.6%), resolved HCV infection in two (3.5%), possible acute HCV infections in nine (15.8%) and likely chronic HCV infections in 32 (56.1%) patients. HCV core Ag was undetectable in 14/15 (93.3%) specimens that tested negative for HCV RNA whereas it was quantified in 34 (out of 39, 87.2%) samples that tested positive for HCV RNA.

**Conclusions:**

Our study provides comprehensive guidance for HCV testing in Gabon, and will help greatly clinicians to improve case definitions for both the notification and surveillance of HCV in patients co-infected with HIV.

## Introduction

Although the diagnosis and antiretroviral treatment (ART) of human immunodeficiency virus (HIV) infection have been improving in resource-limited settings (RLS) for more than a decade, hepatitis C infection remains a largely neglected pandemic. The hepatitis C virus (HCV) is a major health burden. Approximately 170–180 million people worldwide are infected with HCV, most of whom live in RLS [1–3]. HCV is also a prevalent co-infection, and affects chronically about 5.5 million individuals infected with HIV, including around 2.5 million people who live in low and lower-middle-income countries, especially in South East Asia and Africa.

In Africa, efforts to combat hepatitis C are relatively limited (except in some countries like Egypt) [4], because of poor access to HCV care and the numerous difficulties associated with the establishment of HCV status. Indeed, false reactivity in anti-HCV antibody (Ab) tests occurs frequently with third generation (G3) HCV enzyme-linked immunosorbent (ELISA) assays, notably with African specimens [5–7]. The signal-to-cutoff (S/CO) ratio obtained with G3 assays may be increased to overcome this limitation (from ≥ 1.0 to assay-specific values ranging from ≥ 3.8 to ≥ 11.0) [8,9]. Unfortunately, not all HCV ELISA assays have been evaluated and most assessments have been carried out in immunocompetent individuals [5,6,10–14]. In contrast with HIV infection, no rapid point-of-care (POC) tests have been thoroughly evaluated for HCV infection, and such tests cannot be used alone to screen for HCV infection, particularly in HCV/HIV-co-infected patients [15–17]. In addition, the presence of anti-HCV Ab does not necessarily signify active HCV infection. Active HCV disease is often asymptomatic; therefore, many African patients who test positive for anti-HCV Ab receive no additional testing, such as that for HCV RNA and/or HCV core antigen (Ag). In RLS, HCV RNA testing is seldom performed (due to the high cost of commercial viral load [VL] assays) [18], whereas HCV core Ag is rarely used as a marker of viremia [19]. Patients with active HCV disease test positive for HCV RNA and/or HCV Ag and require antiviral treatment [20]. By contrast, individuals who test negative for HCV RNA (or Ag) but positive for HCV Ab are not infectious and do not need HCV treatment because this combination indicates either a past, resolved HCV infection (sustained viral clearance), or false reactivity in HCV Ab assays [21,22]. Finally, acute and chronic HCV infections remain difficult to distinguish, although immunoblotting [23], fluctuations of HCV RNA VL [24] or the avidity of IgG Ab for HCV [25] have been successfully explored for this purpose.

Here, we describe an appropriate method to screen for HCV co-infection in HIV-positive adult patients from Gabon, where HCV false positive and false negative results may be common. We used a combined Ag-Ab G4 ELISA test and determined the optimal S/CO ELISA ratio to identify patients with an active hepatitis C infection, and we compared the results obtained with those of a qualitative HCV RNA reverse-transcriptase PCR (RT-PCR) technique. We also examined whether a recombinant immunoblot assay and a HCV core Ag test were useful to determine HCV infection status, which we divided into four categories: false reactivity, past/resolved infection, early/acute infection and chronic infection.

## Methods

### Patient population and sample processing

We conducted a cross-sectional study using plasma specimens obtained between March 2010 and January 2013 from 762 adults infected with HIV-1, who were followed-up in an outpatient HIV care center located at Franceville (South-East Gabon) [26]. CD4^+^ T-cell counts (CD4) were performed for all subjects at the time of sampling. Our study was approved by the National Ethics Committee of Libreville (N° PROT 0021/2013/SG/CNE). All subjects provided written informed consent for specimen storage and future laboratory studies.

Whole blood specimens were collected in EDTA-containing tubes and processed within 6 hours at the Centre International de Recherches Médicales de Franceville (CIRMF) retrovirology laboratory. After centrifugation, plasma was frozen at -80°C.

### HCV laboratory tests

All plasma samples were initially tested for HCV with the G4 Monolisa HCV Ag-Ab ULTRA ELISA assay (Bio-Rad, Marnes-la-Coquette, France), according to the manufacturer's instructions. This assay is based on the simultaneous detection of HCV core Ag and anti-HCV Ab and can be useful for the detection of recent HCV infections [27]. Specimens with an S/CO ratio < 1.0 were considered non-reactive, and those with an S/CO ratio ≥ 1.0 were considered reactive.

All specimens with an S/CO ratio ≥ 1.0 and 95 randomly selected samples with an S/CO < 1.0 (i.e. negative) were further tested with a qualitative in-house nested HCV RT-PCR assay. This assay targets a highly conserved domain within the non-coding (NC) (5' UTR) region of the HCV genome, as described previously by Bukh *et al*. [28] and is suitable for the detection of HCV genotype 4 which is the most common HCV genotype in Gabon [29].

Finally, specimens with sufficient plasma volume and an S/CO ratio ≥ 1.0 were further tested with two additional assays: the INNO-LIA HCV Score assay (Innogenetics N.V., Gent, Belgium) [30], which assesses anti-HCV Ab profiles, and the Architect HCV Ag assay (Abbott Diagnostics, Wiesbaden, Germany) which measures HCV core Ag levels [31]. For the measurement of HCV core Ag, all samples were diluted 1:10 because of limited plasma volume. Values < 3.0 fmol/l, (equivalent to 700–1100 IU/ml of HCV RNA) were considered ‘not reactive’.

### HCV infection status criteria

We decided to use the serological results obtained by INNO-LIA to distinguish acute from chronic HCV infection and we applied an approach similar to that used for identifying HIV seroconversion by western blotting [32]. We selected this assay because positive or inconclusive results may be associated with false positive ELISA reactions. Therefore, immunoblot assays such as INNO-LIA can be very useful to assess false positive signals, meaning that no further testing is needed. Furthermore, indeterminate profiles (i.e. one isolated band) or weakly positive patterns (only two bands with weak reactivity ≤ 1+) can be encountered during the early phase of HCV infection (recent seroconversion) or in HCV RNA-negative patients who experience a favorable course of HCV infection [30,33,34].

Consequently, patients found positive by G4 ELISA (S/CO ratios ≥ 1.0) were categorized into the four following classes: (i) ‘certain’ false reactivity in ELISA (group 1), which comprised patients who were found negative with INNO-LIA and tested negative for HCV RNA; (ii) ‘certain’ resolved infection (group 2), which comprised patients who were found positive by INNO-LIA but tested negative for HCV RNA; (iii) ‘possible’ acute HCV infection (group 3), which comprised those who were both HCV RNA positive and showed an indeterminate or weakly positive INNO-LIA; and (iv) ‘likely’ chronic HCV infection (group 4), which comprised those who were both HCV RNA positive and exhibited a positive INNO-LIA profile.

### Statistical analysis

A χ2 test was performed to compare the distribution of categorical variables and a non-parametric *Mann-Whitney* test was used to perform intergroup comparisons of continuous variables. P-values of < 0.05 were considered to be significant.

A bi-normal receiver operating characteristic (ROC) curve analysis was conducted to evaluate the performance of the G4 ELISA assay for the prediction of active HCV infection [35]. The results were compared to those of HCV RT-PCR, which was considered the gold standard method for the detection of active HCV infection. The S/CO ratio that gave the highest sensitivity and a specificity of no less than 0.90 was considered to be the ‘optimal’ S/CO ratio. Negative and positive predictive values were calculated taking into account variations in HCV prevalence according to age group (i.e. 18–29, 30–39, 40–49 and ≥ 50 years) [36].

## Results

### Patients' characteristics

Of the 762 patients tested, 67 (8.8%) tested positive for HCV infection with the G4 ELISA assay, by using a S/CO threshold ≥ 1 in accordance with the manufacturer's instructions (Table 1). Anti-HCV Ab positivity was strongly associated with age (p < 10^–6^), but was not dependent on sex. Median CD4 counts were 287 and 339 cells/ in HCV-seropositive and HCV-seronegative subjects, respectively (p = 0.37). Around two thirds of patients were treated with ART (507/762, 66.5%), with no significant difference between HCV-seropositive and HCV-seronegative patients.

**Table 1:** Patient characteristics; entries are n (%) unless otherwise stated.

		HCV-seropositive		HCV-seronegative		p-value^†^	p-value^‡^
	All patients	S/CO ratio ≥ 1	S/CO ratio ≥ 1.7	S/CO ratio ≥ 1	S/CO ratio ≥ 1.7		
n =	762	67	56	695	706		
Female gender	560 (73.5)	47 (70.1)	40 (71.4)	513 (73.8)	520 (73.7)	0.52	0.72
**Age group** (yrs)						< 10^–6^	< 10^–6^
18–29	94 (12.3)	5 (7.5)	4 (7.2)	89 (12.8)	90 (12.7)		
20–29	249 (32.7)	9 (13.4)	5 (8.9)	240 (34.5)	244 (34.6)		
30–39	258 (33.9)	16 (23.9)	13 (23.2)	242 (34.8)	245 (34.7)		
40–49	161 (21.1)	37 (55.2)	34 (60.7)	124 (17.9)	127 (18.0)		
≥ 50							
**CD4** (cells/mm^3^) Median (IQR)	337 (178–499)	287 (156–477)	282 (152–475)	339 (179–499)	337 (178–499)	0.37	0.30
ART						0.67	0.94
No	255 (33.5)	24 (35.8)	19 (33.9)	231 (33.2)	236 (33.4)		
Yes	507 (66.5)	43 (64.2)	37 (66.1)	464 (66.8)	470 (66.6)		
Type of ART						0.62	0.99
ZDV+3TC+EFV or NVP	259 (51.1)	25 (58.1)	19 (51.4)	234 (50.4)	240 (51.1)		
D4T+3TC+EFV or NVP	205 (40.4)	15 (34.9)	15 (40.5)	190 (41.0)	190 (40.4)		
Others	43 (8.5)	3 (7.0)	3 (8.1)	40 (8.6)	40 (8.5)		

^†^P-values were calculated for S/CO ratio ≥ 1.

^‡^P-values were calculated for S/CO ratio ≥ 1.7.

Abbreviations: S/CO, signal-to-cutoff; IQR, interquartile range; ART, antiretroviral treatment; ZDV, zidovudine; D4T, stavudine; 3TC, lamivudine; EFV, efavirenz; NVP, nevirapine.

### Optimal S/CO ratio and performance of the combined Ag-Ab ELISA test in comparison with the HCV RNA RT-PCR test

Of the 67 HCV-seropositive patients, 47 (70.1%) also tested positive for HCV RNA; thus, the overall prevalence of active HCV infection was 6.2% (95% confidence interval (CI) 4.6–8.0%). All 95 patients with S/CO values < 1 tested negative for HCV RNA by RT-PCR.

**Figure 1:**
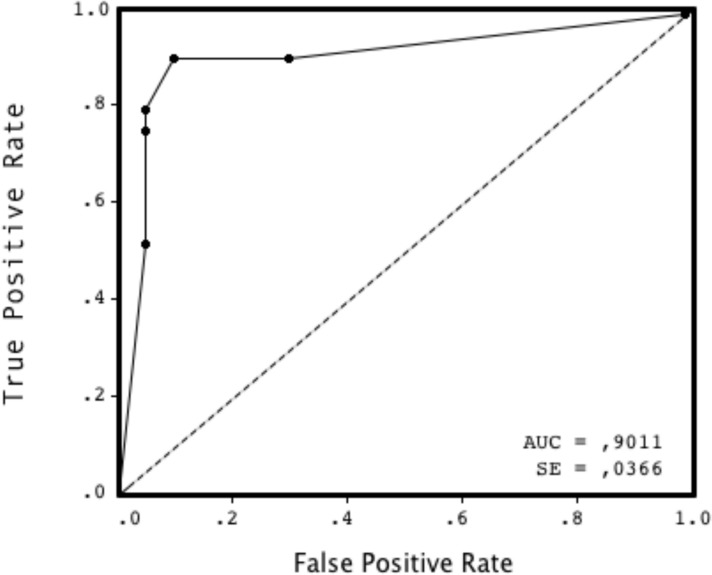
Receiver operating characteristic (ROC) curve for the signal-to-cutoff (S/CO) ratio of the Monolisa HCV Ag-Ab ULTRA ELISA assay (Bio-Rad) for the prediction of active (positive HCV RNA) hepatitis C infections. The area under the ROC curve (AUC) was 0.90.

The ROC curve of the G4 ELISA for the prediction of active (RNA +) hepatitis C is shown in Fig. 1. The area under the ROC curve (AUC) was 0.90 (standard error, 0.04). The optimal S/CO to screen accurately for active HCV infection was determined to be 1.7, which yielded a HCV seroprevalence rate of 7.3% (56/762) (95% CI 5.6–9.4%). At a S/CO threshold ≥ 1.7, patients' characteristics and differences between HCV seropositive and seronegative patients were quite similar than those obtained with a S/CO ≥ 1.0 (see Table 1). At a S/CO of 1.7, the sensitivity of the G4 HCV ELISA technique was 97.9% (46/47, 95% CI 90.0–99.9%) whereas the specificity was 91.3% (105/115, 95% CI 85.0–95.5%). For 18–29, 30–39, 40–49 and ≥ 50 age groups, the positive predictive values were 38.6%, 29.6%, 42.6% and 77.1%, respectively, and the negative predictive values were 99.9%, 99.9%, 99.8% and 99.3%, respectively. The proportion of patients showing an S/CO < 1.7 was similar between those exhibiting strong immunosuppression (CD4<350/mm^3^) and those with a CD4^+^ count ≥ 350/mm^3^ (5/37, 13.5% versus 6/30, 20%, respectively) (p = 0.70).

### Usefulness of the HCV recombinant immunoblot and HCV core Ag assays

**Figure 2:**
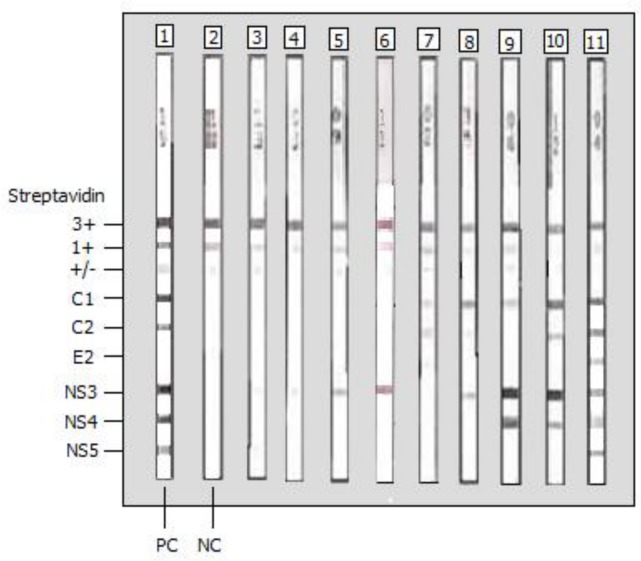
Recombinant immunoblot analysis (the INNO-LIA HCV score assay from Innogenetics) of sera from nine Gabonese HCV/HIV co-infected patients who tested positive for HCV RNA. Lane 1, HCV-positive control; lane 2, HCV-negative control; lanes 3 to 6, sera from four Gabonese HCV/HIV-1-co-infected patients with a faint (+/- or 1+) or strong (3+) isolated NS3 band (indeterminate profile); lane 7, serum from one Gabonese HCV/HIV-1-co-infected patient exhibiting C1 (1+) and C2 (1+) bands but no reactivity to E2, NS3, NS4 and NS5 (weakly positive profile); lane 8, serum from one Gabonese HCV/HIV-1-co-infected patient exhibiting C1 (3+), C2 (+/-) and NS3 (2+) bands but no reactivity to E2, NS4 and NS5 (positive profile); lane 9, serum from one Gabonese HCV/HIV-1-co-infected patients exhibiting strong (4+) reactivity to NS3 and NS4, weak (1+) reactivity to C1 but no reactivity to C2, E2 and NS5; lane 10, serum from one Gabonese HCV/HIV-1-co-infected patients exhibiting strong (3+ or 4+) reactivity to C1, C2, NS3, and NS4 but no reactivity to E2 and NS5; lane 11, serum from one Gabonese HCV/HIV-1-co-infected patient with a complete profile.

Of the 67 specimens found positive by ELISA, 57 and 54 were further tested by HCV INNO-LIA and HCV core Ag assays, respectively, with the following results:

- Fourteen samples (14/57, 24.6%) were found negative by INNO-LIA and tested negative for HCV RNA, indicating false reactivity in the G4 ELISA assay (median S/CO ratio = 1.5, range, 1–3.6). Of these, 13 (one sample was not analyzed due to insufficient volume) also tested negative for HCV core Ag.
- Thirty-seven samples (37/57, 64.9%) were found positive by INNO-LIA. All but two specimens tested positive for HCV RNA. Of these 37 samples, 32 tested positive for HCV core Ag whereas four tested negative (one specimen was not analyzed). One of the two HCV RNA (-) specimens (patient #1678) was HCV core Ag (-); it had an S/CO ELISA ratio of 1 and a weakly positive INNO-LIA profile, indicating a resolved HCV infection. The other HCV RNA (-) specimen (patient #1486) was Ag (+) with a high (= 7) S/CO ELISA ratio and a strong positive INNO-LIA pattern; however, HCV RT-PCR could not be repeated for this sample (insufficient volume). Finally, three specimens (patients #2491, #1595 and #1129) were HCV RNA (+)/Ag (-). There was a wide variety of positive INNO-LIA profiles, with an overall trend of positive correlation between the number and intensity of bands and S/CO ratio in ELISA. The most common reactive bands were NS3 and C1, followed by C2 and NS4 bands, whereas E2 and NS5 were rare (Fig. 2). The different positive INNO-LIA profiles are given in S1 Table.
- Six samples (6/57, 10.5%) showed an indeterminate INNO-LIA profile, including five with an isolated NS3 band and one with an isolated C1 band (Fig. 2) (see also S1 Table). All tested positive for HCV RNA. Three of these samples tested positive for HCV core Ag, two tested negative and one was excluded from testing due to insufficient volume.

### Classification of patients according to HCV infection status

The application of our diagnostic criteria (see material and methods) to the 57 HCV seropositive patients with available INNO-LIA results led to the identification of false reactivity in 14 patients (24.6%), two resolved HCV infections (3.5%), nine possible acute HCV infections (15.8%), and a majority (n = 32) (56.1%) of likely chronic HCV infections. Patients with possible acute HCV infections were younger than those with likely chronic HCV infections (median age, 47 yrs (range, 29–63 years) *vs*. 58 yrs (range, 32–80 years) respectively)) (p = 0.03).

HCV core Ag was undetectable in all but one of the 15 specimens (14/15, 93.3%) that tested negative for HCV RNA (groups 1 and 2). HCV core Ag was quantified in 34 (out of 39, 87.2%) specimens that also tested positive for HCV RNA (groups 3 and 4).

**Figure 3:**
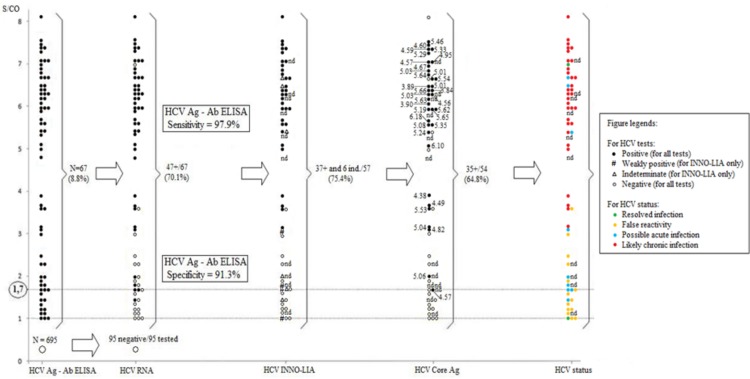
Results of HCV G4 ELISA, qualitative in-house HCV RNA RT-PCR, INNO-LIA and HCV core Ag in 762 HIV-co-infected patients from Franceville, Gabon (2010–2013). Concentrations of HCV core Ag are expressed in log_10_ IU/l. Abbreviations: nt, not done.

All results obtained for each patient are summarized in Fig. 3.

## Discussion

The real burden of HIV/HCV co-infection is unknown in Central Africa due to the very low rate of screening. Our study conducted in Gabon reveals that HCV co-infection is highly prevalent among HIV-1 positive adults. Consistent with previous findings [37–39], around 70% of Gabonese HCV/HIV-co-infected patients fail to control HCV infection and are Ab (+)/RNA (or Ag) (+). The remaining 30% show mainly false reactivity or clear the virus spontaneously, with no detectable HCV RNA (or Ag) in serum in either case.

We found that an S/CO G4 HCV ELISA ratio of 1.7 was optimal to distinguish, with excellent sensitivity and good specificity, between HIV-positive patients who had an active HCV infection and those who did not. Since our study was completed (March 2014), we have been using a S/CO threshold of 1.7 routinely in our laboratory for the serological screening of HCV infection in all specimens. This value is used regardless HIV status of patients that can be known (i.e. positive or negative) or more frequently unknown. The low value of this S/CO ratio (close to 1.0) suggests a weak anti-HCV Ab response in some HIV patients with active HCV infections. As previously reported by others [40], HIV-related immunodeficiency, which is associated with low CD4 counts, may lead to a weak anti-HCV Ab response. Low concentrations of HCV neutralizing Ab have also been reported in HIV/HCV co-infected patients with normal CD4^+^ T-cell counts [41].

The selection of an S/CO threshold is also important to obtain an accurate estimate of the prevalence of HCV in the population. For example, Njouom and colleagues used an S/CO ratio threshold of > 6.0 to examine the prevalence of HCV with the Bio-Rad G3 HCV ELISA assay in Gabonese individuals of unknown HIV status [29]. If we had used this value, without first evaluating the Bio-Rad G4 ELISA assay, the seroprevalence of HCV would have been erroneously reported as 3.2% (25/762), instead of 7.3%.

Our study is among the very few to describe the possible occurrence of acute HCV among HIV-positive patients from sub-Saharan Africa. The use of a combined Ag-Ab ELISA technique and a subsequent recombinant immunoblot assay was a valuable diagnostic strategy because it identified nine patients with indeterminate (n = 6) or weakly positive (n = 3) profiles, indicating possible early/acute HCV infection. The HIV-positive patients included in this study were not men having sex with men (MSM) or injecting drug users (IDUs); thus, medical procedures with contaminated supplies are the most likely source of HCV infection in this population, because HIV-positive patients have high health care-related exposure [42]. HCV transmission between heterosexual partners is also a possibility, at least among younger and sexually active patients, especially given that HIV may facilitate the genital mucosal transmission of HCV [43].

Direct HCV assays are urgently required in RLS. The HCV core Ag assay used in our study has many advantages: this test is not prone to sample carryover contamination, and is both easy to use and relatively cheap, in comparison with quantitative molecular HCV assays [22,44–46]. Nonetheless, we obtained five samples which were Ag-/RNA+, probably due to the 1:10 dilution that was performed prior the measurement of HCV core Ag concentrations. Thus, the sensitivity and specificity of the HCV core Ag assay for the determination of HCV status, as well as its utility for treatment monitoring, merit further evaluation in RLS.

Several limitations need to be acknowledged. First, all patients were already positive for anti-HCV Ab at presentation, and specimens taken before were unavailable, notably for patients with indeterminate or weakly positive immunoblot profiles. In addition, information about alanine transaminase (ALT) levels obtained at the time of sampling was missing from patient files; therefore, we were unable to determine with certainty whether the patients were in the acute phase of HCV infection. Second, we did not include a control group of patients infected with HCV alone because our study was conducted from an HIV care center. Third, the HCV RT-PCR test used in this study was a qualitative tool. Hence, we were unable to compare and to correlate concentrations of HCV core Ag with HCV RNA VL in paired plasma specimens.

In conclusion, it is essential to obtain an accurate picture of the magnitude of the HCV epidemic in Africa, including that in remote health care settings such as ours, in order to provide an appropriate medical response. Similar validation studies are still required in other RLS to define the best practices for detecting HCV viremia and for distinguishing between resolved HCV infections and false positivity in HCV Ab assays in individuals without detectable HCV RNA. The outcome of these studies will provide comprehensive guidelines for reliable testing and clinical management, and will improve case definitions for disease notification and surveillance of HCV in Africa. HCV screening strategies involving both a combined Ag-Ab ELISA and a recombinant immunoblot should be the method of choice in African reference laboratories, at least among immunocompromised HIV-infected patients, who frequently present with either chronic HCV infections, or to a lesser extent, early/acute HCV infections.

## Supporting Information

S1 Table Indeterminate, weakly positive and positive INNO-LIA HCV profiles obtained in 43 Gabonese patients co-infected with HCV/HIV. (DOCX)
