# Supplementary material for: Usefulness of a Fourth Generation ELISA Assay for the Reliable Identification of HCV Infection in HIV-Positive Adults from Gabon (Central Africa)
Source: PLoS One. 2015 Jan 24;10(1):e0116975. doi: 10.1371/journal.pone.0116975 (PMC4305295; doi:10.1371/journal.pone.0116975)
Supplement: S1 Table — (DOCX) [file pone.0116975.s001.docx]

| N° | S/CO | HCV RNA/Ag | INNO-LIA HCV results | | | | | | |
| --- | --- | --- | --- | --- | --- | --- | --- | --- | --- |
|  |  |  | C1 | C2 | E2 | NS3 | NS4 | NS5 | Interpretation |
| 2235 | 1.4 | +/nd | - | - | - | +/- | - | - | IND |
| 2210 | 1.7 | +/+ | - | - | - | 1+ | - | - | IND |
| 228 | 2.0 | +/+ | - | - | - | +/- | - | - | IND |
| 1594 | 5.4 | +/- | - | - | - | 3+ | - | - | IND |
| 3008 | 6.5 | +/+ | 1+ | - | - | - | - | - | IND |
| 2363 | 6.7 | +/- | - | - | - | +/- | - | - | IND |
| 1678 | 1.0 | -/- | +/- | - | - | +/- | - | - | w POS |
| 2491 | 1.7 | +/- | - | +/- | - | +/- | - | - | w POS |
| 1299 | 1.8 | +/nd | 1+ | 1+ | - | - | - | - | w POS |
| 2376 | 3.1 | +/+ | +/- | - | - | +/- | - | - | w POS |
| 2073 | 3.2 | +/+ | 2+ | 1+ | - | 1+ | - | - | POS |
| 1281 | 3.6 | +/+ | 2+ | +/- | - | +/- | - | - | POS |
| 2020 | 3.7 | +/+ | 2+ | +/- | +/- | +/- | - | - | POS |
| 888 | 3.9 | +/+ | - | - | - | 2+ | 1+ | +/- | POS |
| 1595 | 5.0 | +/- | 2+ | +/- | - | 2+ | - | - | POS |
| 9 | 5.1 | +/+ | 2+ | - | - | 4+ | 4+ | - | POS |
| 2744 | 5.4 | +/+ | 2+ | 2+ | +/- | 4+ | 2+ | - | POS |
| 1066 | 5.6 | +/+ | 3+ | - | 3+ | 4+ | 3+ | - | POS |
| 514 | 5.6 | +/+ | +/- | +/- | - | 4+ | - | - | POS |
| 90 | 5.7 | +/+ | 3+ | +/- | - | 3+ | - | - | POS |
| 302 | 5.9 | +/+ | 4+ | 4+ | 2+ | +/- | +/- | - | POS |
| 1670 | 5.9 | +/+ | 2+ | 2+ | +/- | 4+ | - | - | POS |
| 910 | 6.0 | +/+ | 3+ | 3+ | - | 4+ | - | - | POS |
| 1182 | 6.1 | +/+ | 3+ | 3+ | - | 3+ | - | - | POS |
| 349 | 6.2 | +/+ | +/- | - | - | 4+ | - | - | POS |
| 1385 | 6.3 | +/+ | 2+ | 2+ | - | 4+ | 4+ | - | POS |
| 1484 | 6.3 | +/+ | 4+ | 3+ | - | 4+ | 4+ | - | POS |
| 71 | 6.4 | +/+ | 3+ | 3+ | 2+ | 2+ | 2+ | 2+ | POS |
| 985 | 6.4 | +/+ | - | - | - | 4+ | 2+ | - | POS |
| 1067 | 6.5 | +/+ | - | - | - | 4+ | 4+ | 2+ | POS |
| 726 | 6.7 | +/+ | 4+ | 3+ | 3+ | 1+ | 1+ | - | POS |
| 2049 | 6.7 | +/+ | - | 1+ | - | 3+ | 2+ | - | POS |
| 1363 | 6.8 | +/+ | 3+ | 2+ | - | 4+ | 2+ | - | POS |
| 1430 | 6.9 | +/+ | 2+ | 1+ | +/- | 4+ | 2+ | - | POS |
| 1486 | 7.0 | -/+ | 4+ | 3+ | - | 4+ | 3+ | - | POS |
| 254 | 7.1 | +/+ | 4+ | 4+ | - | 4+ | 3+ | - | POS |
| 2202 | 7.1 | +/+ | 4+ | 4+ | - | 4+ | 1+ | - | POS |
| 1609 | 7.3 | +/+ | 3+ | 1+ | - | 4+ | 4+ | - | POS |
| 168 | 7.4 | +/+ | 3+ | 3+ | - | 3+ | - | - | POS |
| 2306 | 7.4 | +/+ | 3+ | - | - | 4+ | 2+ | - | POS |
| 1488 | 7.5 | +/+ | 2+ | 2+ | 2+ | 4+ | 2+ | - | POS |
| 973 | 7.6 | +/+ | 2+ | 2+ | - | 4+ | 2+ | 2+ | POS |
| 1129 | 8.1 | +/- | - | - | - | 4+ | 2+ | - | POS |

Abbreviations: S/CO, ELISA signal-to-cutoff ratio; IND, indeterminate; w POS, weakly positive; POS, positive.
